# Risk of solid cancer in patients with mast cell activation syndrome: Results from Germany and USA

**DOI:** 10.12688/f1000research.12730.1

**Published:** 2017-10-26

**Authors:** Gerhard J. Molderings, Thomas Zienkiewicz, Jürgen Homann, Markus Menzen, Lawrence B. Afrin

**Affiliations:** 1 Institute of Human Genetics, University Hospital of Bonn, Bonn, D-53127, Germany; 2 Institute of Pathology Bonn-Duisdorf, Bonn, D-53123, Germany; 3 Division of Internal Medicine, Department of Gastroenterology and Diabetology, Gemeinschaftskrankenhaus Bonn, Bonn, D-53113, Germany; 4 Armonk Integrative Medicine, Armonk, NY, 10504, USA

**Keywords:** mast cell, systemic mast cell activation disease, systemic mast cell activation syndrome, systemic mastocytosis, cancer, melanoma, breast cancer, cervical carcinoma

## Abstract

***Background:***  It has been shown repeatedly that mast cells can promote or prevent cancer development and growth. If development and/or progression of a solid cancer is substantially influenced by mast cell activity, the frequencies of occurrence of solid cancers in patients with primary mast cells disorders would be expected to differ from the corresponding prevalence data in the general population. In fact, a recent study demonstrated that patients with systemic mastocytosis (i.e., a rare neoplastic variant of the primary mast cell activation disease) have increased risk for solid cancers, in particular melanoma and non-melanoma skin cancers. The aim of the present study is to examine whether the risk of solid cancer is increased in systemic mast cell activation syndrome (MCAS), the common systemic variant of mast cell activation disease.

***Methods:*** In the present descriptive study, we have analysed a large (n=828) patient group with MCAS, consisting of cohorts from Germany and the USA, for occurrence of solid forms of cancer and compared the frequencies of the different cancers with corresponding prevalence data for German and U.S. general populations.

***Results:*** Sixty-eight of the 828 MCAS patients (46 female, 22 male) had developed a solid tumor before the diagnosis of MCAS was made. Comparison of the frequencies of the malignancies in the MCAS patients with their prevalence in the general population revealed a significantly increased prevalence for melanoma and cancers of the breast, cervix uteri, ovary, lung, and thyroid in MCAS patients.

***Conclusions:*** Our data support the view that mast cells may promote development of certain malignant tumors. These findings indicate a need for increased surveillance of certain types of cancer in MCAS patients irrespective of its individual clinical presentation.

## Introduction

Systemic mast cell activation disease (MCAD) comprises a heterogeneous group of multifactorial polygenic disorders characterized by aberrant release of variable subsets of mast cell (MC) mediators together with accumulation of either morphologically altered and immunohistochemically identifiable mutated MCs due to MC proliferation (systemic mastocytosis [SM] and mast cell leukemia [MCL]) or morphologically ordinary MCs due to decreased apoptosis (MC activation syndrome [MCAS] or well-differentiated systemic mastocytosis; Table 1; for details, see 1). The various MCAD classes and clinical subtypes represent varying manifestations of a common process of MC dysfunction ^2– 5^ (for details, see Supplementary File 1). While the prevalence of SM in Europeans ranges between 0.3 and 13 per 100,000 ^6– 8^, the prevalence for MCAS may be in the single-digit percentage range (at least in Germany ^9^).

**Table 1.** Classification of systemic mast cell activation disease.

Systemic mast cell activation disease (MCAD)		
Classes	Systemic mastocytosis (SM)	Systemic mast cell activation syndrome (MCAS)
Subtypes	indolent SM smoldering SM aggressive SM SM with associated hematologic neoplasm mast cell leukemia	with hypertryptasemia without hypertryptasemia

MCs are best known for their effector functions in IgE-associated allergic reactions, but they are also involved in a variety of processes maintaining homeostasis or contributing to disease. Studies have shown that MCs accumulate in tumors and their microenvironment, inferring potential for influencing tumor development, tumor-induced angiogenesis, tissue remodeling, and shaping of adaptive immune responses to tumors by release of certain subsets of mediators (e.g., EGF, NGF, PDGF, SCF, angiopoetin, heparin, IL-8, VEGF). Increased accumulation of MCs within tumor environments has been correlated with poor prognosis, increased metastasis, and reduced survival in several types of human cancer, including melanoma ^10– 12^, prostate carcinoma ^13^, pancreatic adenocarcinoma ^14^, squamous cell carcinomas of the esophagus ^15^, mouth ^16^, and lip ^17^, and Merkel cell carcinoma ^18^. Conflicting findings have been reported for lung adenocarcinoma and breast carcinomas: increased numbers of MCs have been shown to correlate with either a good ^19– 21^ or poor ^22, 23^ prognosis in non-small cell lung cancer. In breast carcinomas, most ^24– 27^ but not all ^28, 29^ studies have linked increased MC density to a good prognosis. For prostate cancer and colorectal cancer, MC densities within the tumors seem to be an independent favorable prognostic factor ^30– 35^, whereas high numbers of peritumoral MCs were associated with a poor prognosis ^36, 37^.

Tumorigenic effects of MCs could be pronounced in MCAD, which is characterized by an increased systemic density and activity of MCs. Knowledge of increased risk for cancer in patients with MCAD would first have important implications for counseling and care of these patients and second would support the idea of an involvement of MCs in tumorigenesis. In fact, a recent study demonstrated that SM patients bear increased risk for solid cancers, in particular melanoma and non-melanoma skin cancers ^38^. However, studies of occurrence of solid cancer in the proportionally prevalent MCAD patient group with MCAS patients have not yet been performed. Therefore, the aim of the present study was to analyze a large MCAS patient group, consisting of two roughly equally sized cohorts from Germany and the USA retrospectively, for the occurrence of solid cancers and to compare their frequencies with corresponding prevalences in German and U.S. general populations.

## Methods

### Diagnostic procedures

MCAS was diagnosed per current provisional criteria (for detailed discussion of the criteria, see Supplementary File 1: Table S2). For differential diagnosis, other diseases presenting similar symptoms were ruled out by appropriate assessments, including laboratory testing, imaging, and/or endoscopy. Occurrence of solid cancer was determined from the medical history obtained at the time MCAS was diagnosed. Due to the retrospective character of the study, pathological material of the solid malignancy could not be specifically stained for MCs. Since in MCAD the profile of MC mediator elevations is highly dependent on individual conditions, correlation analysis of mediator levels determined as part of MCAS diagnosis and the occurrence of specific tumor types was not performed.

### Patient characteristics

***German MCAS patients.*** 417 Caucasians presented consecutively to the *Bonn Interdisciplinary Research Group for Systemic Mast Cell Diseases* between May 2005 and May 2016 for diagnostic evaluation, and were assigned a diagnosis of MCAS diagnosed per current criteria ( 39, Table S2). These patients were included in this retrospective study. The presence of MCAS was the only inclusion criterion; there were no exclusion criteria. From the patients´ clinical files, data concerning the occurrence of solid tumors and hematologic neoplasms up to the time of the presentation at our research group were extracted. All data in this study were collected during routine clinical evaluations of MCAS patients who provided informed consent for use of such data in research. Patient information was anonymized prior to analysis. As such, the Ethics Committee of the Medical Faculty of the University of Bonn classified this study as exempt from requiring specific patient consent. This committee also approved the protocol for this study.

The frequencies of solid cancers in the German MCAS patient cohort were compared with the 10-year prevalences of these cancers in the general German population ^40^.

***U.S. MCAS patients.*** The full population of 411 U.S. patients included in this study was comprised of a 296 patient population examined retrospectively (protocol Pro00015852, diagnoses made between November 2008 and September 2012 per current criteria ( Table S2)) and a 115 patient population examined prospectively (protocol Pro00015857, diagnoses made between April 2012 and October 2013 per current criteria ( Table S2)) at a single center (the Medical University of South Carolina). Protocols were approved by the center’s institutional review board (IRB); the retrospective protocol was deemed IRB-exempt, and all prospective subjects provided written informed consent. Eligible patients for the retrospective protocol were all living, and deceased adult (18 years or older) patients who were, or had been, diagnosed with MCAS from the first such diagnosis at MUSC to the opening of the protocol; the accrual goal for the prospective protocol (targeting adult patients clinically suspected of having MCAS and undergoing diagnostic testing for such, and primarily designed to assess differences in monocyte growth factors between MCAS patients with monocytosis vs. healthy control subjects) was set based on expectations (derived from preliminary data) of finding monocytosis in 70% of MCAS patients. 

Retrospective patients were identified through interrogation of the MUSC enterprise data warehouse for patients diagnosed with MCAS; the subjects’ medical records at MUSC served as the sole source of data for the study, and no patient was contacted to obtain additional information. Prospective patients were identified in the course of the principal investigator’s (LBA’s) clinical work and provided written informed consent. All diagnoses met published criteria ^39^ and were made at age 16 or older. Data items abstracted from eligible patients’ medical records included gender, race, age, symptoms, comorbidities, date of MCAS diagnosis, and all results on file of routine complete blood counts, routine chemistry panels, and diagnostic testing for MCAS per published criteria.

The frequencies of solid cancers in the U.S. MCAS patient cohort were compared with the 32-year prevalences of these cancers in the general U.S. population (from Cancer Statistics Review 1975–2013; all races) ^41^.

### Statistical analysis

Our MCAS patient cohorts were not corrected for cancer risk factors, such as smoking, alcohol intake and body mass index, since the data for the German and U.S. population that were used for comparison were also not corrected in that way. Due to the retrospective nature of the study, we could only calculate the prevalence of a given tumor from the medical histories of the patients, not its incidence. Therefore, standardized incidence ratios could not be calculated and differences between the frequencies of tumor occurrence in our patient groups and the corresponding prevalences in the German and U.S. general population were analyzed by means of two-sided χ ^2^ test using GraphPad InStat V3.05. Here, a significance level of α=0.05 was set.

## Results

### Characteristics of the study population

The 828 MCAS patients included in the study showed male:female ratios of about 1:2.5 in both population groups, which did not differ significantly in age distribution ( Table 2). Forty-four MCAS patients had additional associated hematologic neoplasms, most frequently multiple myeloma, JAK2-positive essential thrombocytosis, and chronic lymphocytic leukemia ( Table 2). In these patients there was no comorbidity of a solid cancer.

**Table 2.** Characteristics of the study population, including demographics and associated hematologic neoplasms.

German MCAS study group (n=417)	
Male (n=105)	Female (n=312)
male to female ratio: 1:2.9	
***Age [years]: mean ± SD, median, range***	
45.7 ± 17.1, 46, 12–86	49.7 ± 14.8, 50, 15–85
Plasmacytoma (n=1) Chronic lymphocytic leukemia (n=1)	
U.S. MCAS study group (n=411)	
Male (n=125)	Female (n=286)
Male to female ratio: 1:2.3	
***Age [years]: mean ± SD, median, range***	
54.9 ± 17.1, 43, 21–96	53.6 ± 17.5, 54, 20–96
Chronic lymphocytic leukemia (n=3) Non-Hodgkin’s lymphoma (n=2) Hodgkin’s lymphoma (n=2) Multiple myeloma (n=6) Acute leukemia (n=2) JAK2-positive essential thrombocytosis (n=4)	Chronic lymphocytic leukemia (n=5) Non-Hodgkin’s lymphoma (n=2) Multiple myeloma (n=6) Acute leukemia (n=2) JAK2-positive essential thrombocytosis (n=8)

MCAS, systemic mast cell activation syndrome; SD, standard deviation.

### Prevalence of solid malignant diseases in the MCAS patients

***German patient group.*** Eighteen of 417 MCAS patients (15 female, 3 male) had developed a solid tumor before the diagnosis of MCAS was made ( Table 3). The most frequent tumor was breast cancer in eight patients ( Table 3). The comparison of the frequencies of the malignancies in the MCAS patients with their 10-year prevalence in the German general population revealed in subsets of the MCAS patients a significantly increased prevalence for melanoma (P<0.001), lung cancer (P<0.0001), breast cancer (P<0.003), cervical carcinoma (P<0.0001), cancer of the urinary bladder (P<0.03) and testis (P=0.05) ( Table 3).

**Table 3.** Comparison of the frequencies of solid cancers in the German MCAS patient cohort with the 10-year prevalences of these cancers in the general German population ^40^.

Malignant diseases of the	10-year prevalence in German population	MCAS patients; n=417
**Skin: melanoma**	f, age 0–39 years : 0.06% f, age > 39 years: 0.19% m, age 0–49 years: 0.05% m, age >49 years: 0.21%	(n= 79) 0 (n=233) **1** = 0.43%, P=0.611, ns (n=60) **1** = 1.67%, P=0.0001 (n=45) 0
**Lung**	f, age 0–49 years : 0.01% f, age > 49 years: 0.13% m, age 0–49 years: 0.01% m, age >49 years: 0.47%	(n=139) **1** = 0.72%, P<0.0001 (n=173) 0 (n=60) 0 (n=45) 0
**Stomach**	f, age 0–69 years: 0.05% f, age > 69 years: 0.26% m, age 0–59 years: 0.05% m, age > 59 years: 0.38%	(n=283) 0 (n=28) 0 (n=83) 0 (n=23) 0
**Gut**	f,m, age 0–59: 0.15% f,m, age > 59 years: 1.5%	(n=323) 0 (n=94) 0
**Carcinoid**	f,m	f: 0.7% **2**
**Breast**	0–39 years: < 0.01% > 39 years: 1.7%	(n=79) **1** = 1.3%, P=0.0025 (n=233) **7** = 3.00%, P=0.0019
**Uterus:** **cervix** **body**	0–39 years: 0.04% > 39 years: 0.16% 0–39 years: 0.05% > 39 years: 0.57%	(n=79) **1** = 1.30%, P<0.0001 (n=233) **1** = 0.43%; P=0.4613,ns (n=79) 0 (n=233) 0
**Prostate**	age 0–59 years: 0.1% age >59 years: 4.2%	(n=82) 0 (n=23) 0
**Testis**	0–39 years: 0.11% > 39 years: 0.09%	(n=42) **1** = 2.4%, P=0.050 (n=63)
**Urinary bladder**	f, age 0–59 years: 0.02% f, age > 59 years: 0.31% m, age 0–59 years: 0.05% m, age > 59 years: 1.68%	(n=236) **1** = 0.42%, P=0.022 (n=76) 0 (n=82) 0 (n=23) 0
**Thyroid gland**	F, all ages : 0.10% M, all ages: 0.32%	(n=312) 0 (n=105) **1** = 0.2%, P=0.7780, ns

Parentheses, number of patients in the respective age group; bold print, number of affected patients; f, female; m, male. P, two-sided P value in the Chi-square-test; ns, not significant

***US patient group.*** Sixty-three of the 411 MCAS patients had developed a solid tumor before the diagnosis of MCAS was made ( Table 4), 47 female and 16 male patients. The difference to the numbers of solid tumors listed in Table 4 is due to the fact that some patients had more than one solid tumor. The most frequent tumors were non-melanoma skin cancer and breast cancer ( Table 4). The comparison of the frequencies of the malignancies in the MCAS patients with their 32-year prevalence in the U.S. general population revealed in subsets of the MCAS patients a significantly increased prevalence for lung cancer (P<0.0001), cervical uterine carcinoma (P<0.0001), ovarian cancer (P<0.02), cancer of the urinary bladder (P<0.04) and thyroid cancer (P<0.01) ( Table 4).

**Table 4.** Comparison of the frequencies of solid cancers in the U.S. MCAS patient cohort with the 32-year prevalences of these cancers in the general U.S. population (from Cancer Statistics Review 1975–2013; all races) ^41^.

Malignant diseases of the	32-year prevalence in the U.S. population	MCAS patients; n=411
**Skin: melanoma**	f, age 0–39 years: 0.042% f, age > 39 years: 0.418% m, age 0–49 years: 0.022% m, age >49 years: 0.681%	(n= 69) 0 (n=218) **1** = 0.46%, ns (n=49) 0 (n=77) **1 ** = 1.30%, ns
**Skin: basal cell and** **squamous cell carcinoma**	f, m, all ages: 0.63% ^1^	(n=411) **14** **[7 BCC]** = 1.70% ****
**Lung**	f, age 0–49 years : 0.009% f, age > 49 years: 0.422% m, age 0–49 years: 0.005% m, age >49 years: 0.444%	(n=119) **2** = 1.68%, P<0.0001 (n=168) **3 ** = 1.19%, ns (n=49) 0 (n=77) 0
**Mesothelium**	f, m, all ages: 0.001%	(n=411) **1 ** = 0.24%
**Stomach**	f, age 0–69 years: 0.018% f, age > 69 years: 0.120% m,age 0–59 years: 0.014% m, age > 59 years: 0.166%	(n=228) 0 (n=59) 0 (n=73) 0 (n=53) 0
**Gut: carcinoma**	f,m, age 0–59: 0.10% f,m, age > 59 years: 1.54%	(n=251) 0 (n=162) **3** = 1.23%, ns
**Liver**	f,m, age 0–39: 0.002% f,m, age > 39 years: 0.05%	(n=94) (n=319) **1** = 0.31%, ns
**Breast**	0–39 years: 0.01% > 39 years: 2.68%	(n=69) 0 (n=218) **10 ** = 4.59%, ns
**Uterus:** **cervix** **body**	0–39 years: 0.03% > 39 years: 0.17% 0–39 years: 0.03% > 39 years: 0.76%	(n=69) 0 (n=218) **5 ** = 2.29%, P<0.0001 (n=69) 0 (n=218) **4 ** = 1.84%, ns
**Ovary**	0–39 years: 0.015% > 39 years: 0.184%	(n=69) (n=218) **1 ** = 0.46%, P=0.0192
**Vulva**	epidemiological data not available	**f 74 ys, f 69 ys**
**Prostate**	age 0–59 years: 0.272% age >59 years: 10.608%	(n=73) 0 (n=53) **5 ** = 9.43%, ns
**Testis**	0–39 years: 0.0.063% > 39 years: 0.099%	(n=25) 0 (n=101) 0
**Urinary bladder**	f, age 0–59 years: 0.011% f, age > 59 years: 0.344% m, age 0–59 years: 0.037% m, age > 59 years: 1.442%	(n=178) 0 (n=109) **2 ** = 1.84%, ns (n=73) 0 (n=53) **3** = 5.66%, P=0.0346
**Thyroid gland**	f, all ages : 0.215% m, all ages: 0.062%	(n=287) **5 ** = 1.39%, P=0.0057 (n=126) **1 ** = 0.79%, ns
**Carcinoid**	epidemiological data not available	**f 29 ys, f 75ys**
**Sarcoma**	epidemiological data not available	**f 61 ys** (sarcoma type not specified)

Parentheses, number of patients in the respective age group; bold print, number of affected patients; f, female; m, male, ys, years; BCC, basal cell carcinoma. P, two-sided P value in the Chi-square-test; ns, not significant ^1^Statistics of basal and squamous cell skin cancers are not reported to and tracked by cancer registries. Data based on mathematical modeling are taken from 48.

Raw data supporting the findings in this study Sheet 1, Data for the German patient cohort; Sheet 2, Data for the U.S. patient cohort. Click here for additional data file. Copyright: © 2017 Molderings GJ et al. 2017 Data associated with the article are available under the terms of the Creative Commons Zero "No rights reserved" data waiver (CC0 1.0 Public domain dedication).

## Discussion

It has been shown that MCs can promote tumor development and growth (for references, see *Introduction*). These tumorigenic effects of MCs could be pronounced in MCAD ( Table 1), which is characterized by an increased systemic density and activity of MCs. An increased risk for solid cancer has been demonstrated for SM patients ^38^, but MCAS patients have not been investigated in this respect so far.

The present analysis of the frequencies of solid malignancies in the MCAS patients revealed a significantly increased prevalence of melanoma in a subset (male, <50 years) of the German patient group. The more heterogeneous ethnic makeup of the U.S. patient group might be a factor in that group’s lower rate of melanoma compared to the Caucasian-only German group, as different ethnic groups might have different risk for melanoma. An increased risk for melanoma has been observed in previous studies with Caucasian SM patients ^38, 42– 45^ with a prevalence of 5% in 81 Swedish SM patients ^43^. It has been speculated that, as in SM, mutations in tyrosine kinase *KIT*, which have also been reported in melanoma ^46, 47^, may predispose MCAD patients to melanoma. In addition, the cytokines produced by MCs may recruit melanocytes and stimulate proliferation ^12, 43^.

Furthermore, an increased risk for cervical carcinoma, lung and bladder cancer was found in the present study in both the German and U.S. cohorts, and increased risk for breast carcinoma and cancer of the testis was found in the German cohort. The marginal discordances in the type of solid cancers observed in the two groups could be explained by the more heterogeneous ethnic makeup of the U.S. patient group and the limited number of patients included in the study.

It is striking that it is the skin and respiratory and genitourinary tracts – i.e., environmental interfaces – where the increased frequencies were (mostly) seen. Given that (1) MCs preferentially site themselves at the environmental interfaces, (2) MCs have ample capacity to promote local and systemic inflammatory states, and (3) risk for many forms of cancer appears correlated with chronic inflammation, one wonders if the increased risk for these skin and genitourinary tract cancers bears any relationship to the relatively increased density of MCs under normal circumstances in these sites, and thus also potential for greater chronic inflammatory stimulus in these sites.

In the combined study population, intestinal cancer and prostate cancer were as frequent as in the German and U.S. general population. Thus, the reported protective effect of an increased systemic MC activity and density on the development of these two cancer forms is not observed in our study population who have increased systemic MC activity and density. Our findings are in contrast to data from epidemiological studies suggesting that allergic disease, which is also characterized by increased MC activity, is associated with decreased risk for colorectal cancer ( 48, 49; for review, see 50). It is conceivable that in the present study the ethnic heterogeneity of the combined study population might have masked the expected protective effect ^51^. In addition, the number of patients included in the present study might still be too low to clearly reveal the purported protective effect.

It is a limitation of the present study that, due to our limited numbers of MCAS patients, we had to partition the patients into only two age groups for each type of cancer for statistical analysis, whereas data from the German and U.S. general populations were partitioned into at least five age groups. However, according to the distribution of the prevalence data within those five groups, it was possible to break them down into two age groups for comparison with our frequency data. Moreover, the reliability of our frequency data is supported by recent similar findings in a Danish MCAD cohort consisting of 687 SM patients ^38^. Unfortunately, it was not possible to compare the frequencies of the solid malignancies determined in our MCAS patient cohorts with those in SM because either the respective frequencies were not reported ^38^ or the MCAD variant of the included patients were not defined exactly (i.e., SM or MCAS) ^52^. Moreover, neither of these publications provided the age of the patient at which the cancer occurred. Finally, our German and U.S. cohorts appear to differ in their frequencies of hematologic neoplasms. At present, we could only speculate about reasons for this difference.

In conclusion, our data support the view that MCs may promote development of certain malignancies. These findings indicate a need for increased surveillance of cancers more frequently seen in MCAS patients. Given the influence of inflammation on neoplasia and the chronic multisystem inflammatory state that is the essence of MCAS, it is conceivable that treatment of MCAS (presuming recognition of MCAS in the first place) may reduce risk for neoplasia. It also is possible that treatment of MCAS in the setting of cancer (regardless of whether the MCAS or the cancer is discovered first) may favorably influence the course of the cancer (e.g. 53), similar to the favorable effects often seen when SM is treated in the setting of associated hematologic neoplasia ^54^.

## Data availability

The data referenced by this article are under copyright with the following copyright statement: Copyright: © 2017 Molderings GJ et al.

Data associated with the article are available under the terms of the Creative Commons Zero "No rights reserved" data waiver (CC0 1.0 Public domain dedication). 

**Dataset 1: Raw data supporting the findings in this study.** Sheet 1, Data for the German patient cohort; Sheet 2, Data for the U.S. patient cohort. doi, 10.5256/f1000research.12730.d181450 ^55^

## Supplementary material

**Supplementary File 1: Classification of and diagnostic criteria for mast cell activation disease.**

**Table S1.** WHO criteria defining *Systemic Mastocytosis*
  **Table S2.** Criteria defining *Mast Cell Activation Syndrome*
  **Table S3.** Criteria alternatively proposed to define mast cell activation syndrome
