# Supplementary material for: Risk of solid cancer in patients with mast cell activation syndrome: Results from Germany and USA
Source: F1000Res. 2017 Oct 26;6:1889. [Version 1] doi: 10.12688/f1000research.12730.1 (PMC5710302; doi:10.12688/f1000research.12730.1)
Supplement: Supplementary file 2 [file f1000research-6-13790-s0001.tgz › c5099b3b-55a8-46ee-b508-7b5ec22db741.doc]

**Classification of and Diagnostic Criteria for MCAD**

Classification systems and diagnostic criteria for mastocytosis were first proposed in the 1980s [1, 2]. Since 2001 the World Health Organization (WHO) criteria (Table S1) have guided classification and diagnosis of systemic mastocytosis globally [3]. Recognition of the need for an alternative, mast cell activation (MCAS)-like diagnosis came about in part because of the discovery of patients whose clinical presentation was highly consistent with systemic mastocytosis (SM) but who did not satisfy the major SM diagnostic criterion and only satisfied one or two of the minor criteria. At present several independent international expert groups on MCAD (e.g., [4-11]) have proposed provisional classification schemes and diagnostic criteria for MCAS which are broadly similar but differ in two aspects: (1) Based on negative clonality evaluations of selected KIT sequences some groups (e.g., [4]) differ between clonal and non-clonal MCAS despite the demonstrated great complexity of genetic and epigenetic alterations across the MCAD spectrum [12] (for review, see [13]. (2) Presently there are two principal proposals for diagnostic criteria for MCAS (Tables S2 and S3). The diagnostic criteria for MCAS by some groups (e.g., [4]) are rather restrictive without any scientific justification. Potential problems with the Valent *et al.* criteria [4] (Table S3) include (1) non-recognition of many of the symptoms that can result from MC activation, (2) lack of published validation that the described tryptase increase within the reference range reliably distinguishes ordinary baseline fluctuation of tryptase from fluctuation induced by aberrant MC activation, (3) practical difficulties in providing/obtaining a specimen for serum total tryptase within 4 hours of onset of an exacerbation of symptoms, and (4) practical difficulties in finding - in such a heterogeneous disease - MC-targeting agents that can effect at least partial response. Thus, for example, patients whose aberrant MC activation causes substantial muscle/joint/bone aching, constipation and abdominal pain, paresthesias, adenitis, and cognitive dysfunction [5] but not the symptoms listed by this group would not qualify for the diagnosis. Furthermore, patients often are sufficiently disabled during a flare of symptoms that they cannot easily get to a medical center, and those who do travel to an urgent care facility or emergency department often encounter providers resistant to pursuing tests not needed for immediate care of the presenting symptoms. Such resistance often persists even when the patient presents a prescription from a MC disease specialist specifically requesting MC mediator testing at times of such flares. Finally, in concert with the observed marked heterogeneity of the clinical presentation of MCAS (perhaps due to underlying marked mutational heterogeneity; [12, 13]), some MCAS patients benefit little from the first few or several MC-targeting therapies tried, risking premature rejection of the diagnosis.

In practice, the scheme of the criteria for diagnosis of MCAS (Table S2) used in the present study most often leads to diagnosis of MCAS by pairing of the major criterion with the minor criteria 1, 4 and/or 5, and it appears to permit applying the diagnosis of MCAS – and therefore also pursuing therapy for MCAS – in a wider population of otherwise mysteriously chronically multisystemically ill patients.

As yet there have been no studies of the diagnostic accuracy or efficiency of the different diagnostic schemes which would demonstrate a superiority of one of the diagnostic schemes. In addition, an opinion leadership by one of the expert groups is not authorized by any governmental institution or national and international scientific organization, even if this may be suggested by the name of one or another group of experts.

**Table S1** WHO criteria defining *Systemic Mastocytosis (SM)*

**Major criterion**

Multifocal dense infiltrates of MCs (i.e., aggregates of >15 MCs) in marrow and/or other extracutaneous organ(s)

**Minor criteria**

1. Abnormal spindle-shaped morphology in >25% of MCs in marrow or other extracutaneous organ(s)

2. Abnormal marrow MC expression of CD2 and/or CD25 (i.e., co-expression of CD117/CD25 or CD117/CD2)

3. Activating KIT mutation at codon 816 in MCs in extracutaneous organ(s)

4. Serum total tryptase > 20 ng/ml (does not apply in patients who have associated hematologic non-mast-cell lineage disease)

According to the WHO criteria, the diagnosis *syste­mic mastocytosis* is established if the major criterion and at least one minor criterion or at least three minor criteria are fulfilled [14, 15].

**Table S2** Criteria defining *Mast Cell Activation Syndrome (MCAS)*

**Major criteria**

1. Constellation of clinical complaints attributable to patho­lo­gi­cally increased MC activity (MC ­mediator release ­syndrome)

**Minor criteria**

1. Multifocal or disseminated infiltrates of MCs in marrow and/or extracutaneous organ(s) (e.g., gastrointestinal or genitourinary tract; >19 MCs/high power field)

2. Abnormal spindle-shaped morphology in >25% of MCs in marrow or other extracutaneous organ(s)

3. Abnormal MC expression of CD2 and/or CD25 (i.e., co-expression of CD117/CD25 or CD117/CD2)

4. MC genetic changes (e.g., activating KIT codon 419, 509 or 560 mutations) shown to increase MC activity

5. Evidence (typically from body fluids such as whole blood, serum, plasma, or urine) of above-normal levels of MC mediators including:

- tryptase
- histamine or its metabolites (e.g., *N*-methylhistamine)
- heparin
- chromogranin A (note potential confounders of cardiac or renal failure, neuroendocrine tumors, or recent proton pump inhibitor use)
- other relatively MC-specific mediators (e.g., eicosanoids including prostaglandin (PG) D_2_, its metabolite 11-β-PGF_2α_, or leukotriene E4)

6. Symptomatic response to inhibitors of MC activation or MC mediator production or action

Criteria proposed to define mast cell (MC) activation syndrome when all other diagnoses that could better explain the full range and chronicity of the findings in the case have been excluded (modified from [16]). The diagnosis *mast cell activation syndrome* is made upon fulfilment of the major criterion plus at least one minor criterion.

**Table S3.** Criteria alternatively proposed to define mast cell (MC) activation syndrome [4].

1. Chronic/recurrent symptoms (flushing, pruritus, urticaria, angioedema, nasal congestion or pruritus, wheezing, throat swelling, headache, hypotension, and/or diarrhea) consistent with aberrant MC mediator release.

2. Absence of any other known disorder that can better account for these symptoms.

3. Increase in serum total tryptase of 20% above baseline plus 2 ng/mL during or within 4 h after a symptomatic period.

4. Response of symptoms to histamine H_1_ and/or H_2_ receptor antagonists or other “MC-targeting” agents such as cromolyn.

**References**

1. Travis WD, Li CY, Bergstralh EJ et al. (1988) Systemic mast cell disease. Analysis of 58 cases and literature review. Medicine (Baltimore 67:345-368

2. Travis WD, Li CY, Yam LT et al. (1988) Significance of systemic mast cell disease with associated hematologic disorders. Cancer 62:965-972

3. Valent P, Horny HP, Escribano L et al. (2001) Diagnostic criteria and classification of mastocytosis: a consensus proposal. Leuk Res 25:603-625

4. Valent P, Akin C, Arock M et al. (2012) Definitions, criteria and global classification of mast cell disorders with special reference to mast cell activation syndromes: a consensus proposal. Int Arch Allergy Immunol 157:215-225

5. Afrin LB, Butterfield JH, Raithel M et al. (2016) Often seen, rarely recognized: mast cell activation disease - a guide to diagnosis and therapeutic options. Ann Med 48:190-201

6. Broesby-Olsen S, Dybedal I, Gülen T et al. (2016) Multidisciplinary management of mastocytosis: Nordic expert group consensus. Acta Derm Venereol 96:602-612

7. Pardanani A, Chen D, Abdelrahman RA et al. (2013) Clonal mast cell disease not meeting WHO criteria for diagnosis of mastocytosis: clinicopathologic features and comparison with indolent mastocytosis. Leukemia 27:2091-2094

8. Sánchez-Muñoz L, Morgado JM, Álvarez-Twose I et al. (2016) Diagnosis and classification of mastocytosis in non-specialized versus reference centres: a Spanish Network on Mastocytosis (REMA) study on 122 patients. Br J Haematol 172:56-63

9. Bodemer C, Hermine O, Palmérini F et al. (2010) Pediatric mastocytosis is a clonal disease associated with D(816)V and other activating c-KIT mutations. J Invest Dermatol 130:804-815

10. Pagano L, Valentini CG, Caira M et al. (2008) Advanced mast cell disease: an Italian hematological multicenter experience. Int J Hematol 88:483-488

11. Hamilton MJ, Hornick JL, Akin C et al. (2011) Mast cell activation syndrome: A newly recognized disorder with systemic clinical manifestations. J Allergy Clin Immunol 128:147-152

12. Altmüller J, Haenisch B, Kawalia A et al. (2017) Mutational profiling in the peripheral blood leukocytes of patients with systemic mast cell activation syndrome using next-generation sequencing. Immunogenetics doi: 10.1007/s00251-017-0981-y. Epub ahead of print

13. Molderings GJ. (2015) The genetic basis of mast cell activation disease - looking through a glass darkly. Crit Rev Oncol Hematol 93:75-89

14. Valent P, Horny HP, Li CY et al. (2001) Mastocytosis. In: Jaffe ES, Harris NL, Stein H, Vardiman JW, editors. World Health Organization (WHO) Classification of Tumours. Pathology and Genetics. Tumours of Haematopoietic and Lymphoid Tissues. Lyon: IARC Press; 2001; pp. 291-302

15. Valent P, Akin C, Escribano L et al. (2007) Standards and standardization in mastocytosis: consensus statements on diagnostics, treatment recommen­dations and response criteria. Eur J Clin Invest 37:435-453

16. Molderings GJ, Brettner S, Homann J et al. (2011) Mast cell activation disease: a concise practical guide for diagnostic workup and therapeutic options. J Hematol Oncol 4:10
