# Supplementary material for: Risk of solid cancer in patients with mast cell activation syndrome: Results from Germany and USA
Source: F1000Res. 2017 Oct 26;6:1889. [Version 1] doi: 10.12688/f1000research.12730.1 (PMC5710302; doi:10.12688/f1000research.12730.1)
Supplement: Peer review history

# Peer review: Referee response for version 1

**DOI:** 10.5256/f1000research.13790.r28307

**Published:** 2017-11-30

**Authors:** Leonard B. Weinstock

**Affiliations:** 1 Specialists in Gastroenterology, LLC, Washington University School of Medicine, St. Louis, MO, USA

The mast cell (MC) was discovered by **Paul Ehrlich** in 1876. The MC is a unique bone marrow-derived cells since mast cells (MCs) are not found in the blood stream. They are located in tissues of the body where they can reside for many months or years.

 The first historical MC path led to the understanding of its role in health. The next path of MC research led to the discovery of the rare, yet well accepted disease mastocytosis - a primary malignant process that had systemic MC-mediator induced symptoms. The next remarkable discovery was that mutations could lead to active MCs and be responsible for over 48 symptoms throughout the body. This confounding presentation of mast cell activation syndrome (MCAS) usually causes confusion and frustration to the physician as well as to the patient. Once MCAS is diagnosed, treatment is often helpful and allows most patients to regain a better quality of life.

 The next query posed by Molderings et al is whether or not the MC plays a role in cancer in humans. There are four animal models to suggest that there was a modulating effect in cancer development. Other studies show potential suppression of cancer by MCs. The human connection has been explored in this journal in a large population of MCAS patients. In this study, 828 MCAS were investigated. A history of solid malignant lesions was queried prior to establishing the diagnosis of MCAS.

 The authors suggest that this data should be compelling evidence for physicians to perform more intense screening for malignancies in those who have MCAS. The ultimate challenge is to see if modulation, suppression or stabilization of MCs and/or MCAS can decrease the risk and/or progression of cancer.

I have read this submission. I believe that I have an appropriate level of expertise to confirm that it is of an acceptable scientific standard.

# Peer review: Referee response for version 1

**DOI:** 10.5256/f1000research.13790.r27327

**Published:** 2017-11-21

**Authors:** Bastian Walz

**Affiliations:** 1 medius Klinik Kirchheim, Vaskulitiszentrum Süd, Klinik für Innere Medizin, Rheumatologie und Immunologie, Kirchheim-Teck, Germany

They use very large cohorts for the study.

 The authors found an accumulation of hematological neoplasia in the US cohort. This circumstance is probably due to a selection bias, as the attending physician is a haemato-oncologist.

 The accumulation of malignant skin tumours could also be caused by a photosensitisation due to mast cell activation disease, as many patients suffer from a rapid skin reaction to UV light.

 The present work would be proposed for publication, not least because there are still only a few working groups in this area and there is a strong unmet scientific need for further investigation and information in mast cell activation disease.

I have read this submission. I believe that I have an appropriate level of expertise to confirm that it is of an acceptable scientific standard.
